# Role of Bioelectrical Impedance Analysis in Detecting Nutritional Disorders in Institutionalized Psychogeriatric Patients

**DOI:** 10.3390/nu17243839

**Published:** 2025-12-08

**Authors:** Beatriz de Mateo Silleras, Sara Barrera Ortega, Laura Carreño Enciso, Gema Gallego Herreros, Sandra de la Cruz Marcos, Paz Redondo del Río

**Affiliations:** 1 Department of Nutrition and Food Science, Faculty of Medicine, University of Valladolid, 47005 Valladolid, Spain; bmateo@uva.es (B.d.M.S.); gema.gallego@uva.es (G.G.H.); sandra.cruz@uva.es (S.d.l.C.M.); paz.redondo@uva.es (P.R.d.R.); 2 Psycho-Geriatric Area, Assistance Center of San Juan de Dios, 34005 Palencia, Spain; sara.barrera@sjd.es

**Keywords:** bioimpedance, BIVA, malnutrition, sarcopenic obesity, obesity, psychogeriatric patients, body composition

## Abstract

**Background**: Institutionalized older adults often experience cognitive and functional decline and altered body composition (BC), making nutritional assessment difficult. Bioelectrical impedance analysis (BIA) offers a simple and non-invasive method to evaluate BC; classic and specific bioelectrical impedance vector analysis do not require predictive models or assumptions about hydration status. **Objective**: This study aimed to evaluate the utility of BIA, classic bioelectrical impedance vector analysis (BIVA), and specific BIVA (BIVA-Sp) in detecting nutritional and other related disorders in institutionalized psychogeriatric patients. **Methods**: A cross-sectional study was conducted in 95 institutionalized older adults (52 men, 43 women; mean age: 80 years). Clinical and functional data, including frailty, dependency, handgrip strength, and anthropometry, were collected. BC was assessed using BIA. Nutritional diagnoses included malnutrition (GLIM criteria), sarcopenia (EWGSOP2), adiposity, and sarcopenic obesity (SOGLI criteria). Mean impedance vectors and 95% confidence ellipses were generated for BIVA and BIVA-Sp. Individual vectors were compared with reference data from healthy older adults. Statistical analyses compared clinical variables and impedance vector distributions between groups. **Results**: Classic BIVA differentiated patients with sarcopenia and sarcopenic obesity, while BIVA-Sp identified vector shifts associated with adiposity and sarcopenic obesity. Neither BIVA nor BIVA-Sp discriminated patients based on body mass index or malnutrition status. **Conclusions**: The application of BIVA in institutionalized psychogeriatric patients allows for easier, faster, and more effective detection of changes in BC and hydration status compared with conventional methods. This enables individualized monitoring and facilitates interventions that may reduce complications, functional decline, and hospitalizations, thereby improving their quality of life.

## 1. Introduction

Institutionalized older adults, with or without dementia, frequently present malnutrition (MN), a condition associated with functional decline, disability, frailty, sarcopenia, reduced physical performance, muscle mass and strength loss, poorer quality of life, and increased morbidity and mortality [1]. Obesity [2], sarcopenia [3], and sarcopenic obesity (SO) [4,5] are also frequently observed in this population. These disorders have important health implications. In older adults, obesity is associated with an increased risk of falls, metabolic disorders, type 2 diabetes, cardiovascular disease, osteoarthritis, and fractures [5]. Sarcopenia has been related to higher all-cause mortality, increased risk of falls and fractures, cognitive impairment, and depression [5]. SO is associated with reduced physical performance and increased all-cause and cardiovascular mortality, as well as multiple comorbidities including cardiovascular disease, stroke, metabolic disorders, cognitive impairment, arthritis, physical disability, and pulmonary disease [4,5]. All these nutrition-related disorders involve body composition (BC) alterations often missed in assessments using routine methods like body weight or conventional bioelectrical impedance analysis (BIA).

Early identification of nutrition-related disorders is essential for guiding therapeutic interventions that may reduce morbidity and mortality and improve quality of life in this vulnerable population. However, the nutritional assessment of long-term institutionalized psychogeriatric patients—often bedridden—can be challenging. Major difficulties arise from a lack of cooperation: cognitive impairment frequently limits effective communication and understanding, including the ability to answer questions or follow instructions. In addition, the frailty and physical and functional dependency of these individuals mean they may require assistance with mobilization and virtually any task. These factors hinder and restrict the performance of tests and measurements that depend on cognitive and/or functional participation. Furthermore, many commonly used indicators of nutritional status are not sensitive to rapid changes, complicating the early detection of nutritional alterations that may influence disease progression and exacerbate underlying conditions, increasing comorbidity, multimorbidity, and mortality. Therefore, simple, accurate, and cost-effective techniques such as BIA and its vector modalities are needed to diagnose and monitor body composition changes in institutionalized older adults.

BIA is a widely used indirect method for assessing BC in clinical settings, valued for its safety, speed, simplicity, non-invasiveness, portability, affordability, and minimal technical demands. It requires minimal patient cooperation and is suitable for bedridden individuals [6], making it an ideal technique for the assessment of psychogeriatric patients. BIA measures tissue resistance to low-intensity alternating electrical currents at varying frequencies [6]. There are different BIA modalities, each with specific applications and interpretations.

Single-frequency BIA, or conventional BIA at 50 kHz, is the most frequently used approach for estimating BC. It estimates fat-free mass (FFM) and total body water using predictive mathematical models based on a two-compartment model and assuming a constant hydration fraction of FFM. However, this assumption is not always valid, particularly in clinical conditions associated with fluid imbalance [7], and hydration of FFM also varies with age [6]. Therefore, it is essential to use predictive equations developed in populations with physiological and pathological characteristics similar to those of the study sample [8]. Bioelectrical impedance vector analysis (BIVA) is a semi-quantitative method that does not rely on mathematical models to estimate the absolute or relative values of body compartments. As such, it is not affected by the aforementioned limitations or by the errors inherent to predictive equations. Classic BIVA enables the assessment of changes in body fluids, hydration status, and body cell mass, by plotted impedance vectors normalized for height on a graph of the normal distribution of a reference population (tolerance ellipses) [9,10,11]. Specific BIVA (BIVA-Sp) adjusts impedance vectors for cross-sectional areas to reduce the effect of body dimensions [12]. BIVA-Sp allows the assessment of changes in fat mass percentage (FM%), and body cell mass [13].

Several studies show BIVA detects subtle BC changes in older adults not captured by conventional BIA [14,15,16]. Additionally, BIVA-Sp offers greater precision than classic BIVA in estimating FM% and extracellular to intracellular water ratio, both in free-living elderly individuals [17] and in institutionalized older adults with dementia [18]. However, few studies have examined the different interpretations of classic and specific BIVA within the same sample of institutionalized psychogeriatric patients. This study aimed to assess the utility of BIA, classic BIVA, and BIVA-Sp in detecting nutritional and other related disorders in institutionalized psychogeriatric patients.

## 2. Materials and Methods

### 2.1. Study Design and Participants

A cross-sectional study was conducted at a psychogeriatric center in Palencia (Spain) in May 2022. No prior sample size calculation was performed, as no specific hypothesis was being tested; instead, the aim was to obtain the largest possible sample. Therefore, all institutionalized subjects aged over 60 years who were free from acute intercurrent illnesses (e.g., respiratory infection, urinary tract infection, influenza, COVID-19), who had no contraindications for bioimpedance analysis (fluid imbalances, amputations, metallic prostheses, or pacemakers), and who met the BIA application criteria [8] [body mass index (BMI) 16–34 kg/m^2^, no abnormal tissue hydration or edema, and no neuromuscular diseases] were included. The center’s geriatrician, a member of the research team, verified residents’ eligibility. All patients who met the inclusion criteria agreed to participate.

### 2.2. Measurements

Clinical and demographic data were extracted from the medical records. The main pathology was diagnosed according to the Diagnostic and Statistical Manual of Mental Disorders, Fifth Edition (DSM-5) [19] and the International Classification of Diseases, 11th Revision (ICD-11) [20]: dementia (major neurocognitive disorder), schizophrenia, and intellectual disability, mainly. Frailty was assessed using the FRAIL test [21], and dependence using the Barthel index [22]. Handgrip strength was measured with a Jamar hydraulic hand dynamometer (Patterson Medical Holdings Inc., Bolingbrook, IL, USA) following the protocol of the American Society of Hand Therapists [23]. Sarcopenia was diagnosed according to the revised criteria of the European Working Group on Sarcopenia in Older People (EWGSOP2) [24]. Nutritional risk was screened using the full version of the Mini Nutritional Assessment (MNA) [25]. MN was diagnosed based on the criteria of the Global Leadership Initiative on Malnutrition (GLIM) [26,27].

Anthropometric measurements (body weight, height or heel–knee distance [28], and body circumferences) were obtained following the NHANES [29] and WHO protocols [30], using standard instruments [SECA (Hamburg, Germany) vertical stadiometer and scale, and a Cescorf non-extensible tape (Porto Alegre, Brasil)]. All measurements were taken by the same examiner (a trained dietitian–nutritionist). Nutritional evaluation was conducted following the protocol of the Spanish Society of Parenteral and Enteral Nutrition (SENPE) and the Spanish Society of Geriatrics and Gerontology (SEGG) [31]. BMI was classified according to WHO cut-off points and the consensus document for older adults [30,32]. Waist circumference was categorized based on WHO cut-offs for visceral obesity [33]. Obesity was defined based on the criteria proposed by Gallagher et al. [34]. According to the recommendations of the Sarcopenic Obesity Global Leadership Initiative (SOGLI) outlined in the ESPEN-EASO consensus [4,35], SO was diagnosed based on (1) handgrip strength cut-off points [24], (2) appendicular skeletal muscle mass (ASMM) normalized by body weight (ASMM/W) [36], and (3) FM% [34].

Whole-body BIA was performed using a tetrapolar electrode configuration in single-frequency mode at 50 kHz with a BIA-101 analyzer (AKERN-Srl, Florence, Italy), following the standard protocol [37]. Participants were positioned in the supine position with their limbs slightly abducted and not touching each other. Electrodes were placed on the right side of the body at the metacarpophalangeal and metatarsophalangeal lines of the hand and foot (injector electrodes), and at the midline between the styloid processes of the wrist and the midline between the ankle malleoli (detector electrodes), after cleaning the sites with ethyl alcohol. Measurements were taken in the morning, at room temperature, after a minimum 4-h fast and bladder emptying; participants were instructed to remain still during the assessment.

Raw electrical variables—resistance (R), reactance (Xc), and phase angle (PhA)—were recorded. BC compartments were estimated as follows: FFM using an equation derived from Caucasian subjects aged 22 to 94 years, validated with DXA (Kyle et al. model) [8]; FM as the difference between body weight and FFM; and ASMM using Sergi et al.’s equation [38]. The fat-free mass index (FFMI), fat mass index (FMI), and appendicular skeletal muscle mass index (ASMMI) were calculated by dividing each corresponding value by height squared (in meters).

For BIVA, the impedance vector components (R and Xc) were standardized by height [R/H (Ohm/m) and Xc/H (Ohm/m)] and plotted on a BIVA graph. Individual impedance vectors were compared to the reference vector distribution for healthy older adults [10,39] using the 50th, 75th, and 95th percentile tolerance ellipses. To compare subgroups according to the criteria used in the study (sex, sarcopenia, MN, SO, etc.), 95% confidence ellipses for mean impedance vectors were plotted using software provided by Piccoli and Pastori [40].

For the BIVA-Sp approach, R and Xc values were corrected (Rsp and Xcsp) by multiplying their values by A/L, where A represents the cross-sectional area (m^2^), estimated as: (0.45 × arm area + 0.10 × waist area + 0.45 × calf area), and L(m) is height multiplied by 1.1. Segmental areas were calculated using the formula C^2^/4π, where C(m) is the circumference of the arm, waist, or calf. The specific values were rescaled by a factor of 100 [12]. Individual specific impedance vectors were compared with the reference vector distribution for the older adult population [39], based on the 50th, 75th, and 95th percentile tolerance ellipses, using the same software employed for classic BIVA. Finally, to compare subgroups, 95% confidence ellipses were plotted for the mean specific vectors of each group.

Complete data were available for all variables for every participant in the study (no missing data). In patients unable to cooperate due to cognitive decline, handgrip strength was evaluated considering everyone’s clinical context and functional capacity (Barthel Index).

### 2.3. Statistical Analysis

Categorical variables were expressed as absolute and relative frequencies [% (n)], and quantitative variables as mean (SD). The normality of quantitative variables was assessed using the Kolmogorov–Smirnov test with Lilliefors’ correction for larger samples and the Shapiro–Wilk test for smaller samples (<30 participants). Differences in quantitative variables between two groups were examined using Student’s *t*-test for independent samples or the Mann–Whitney U test for non-normally distributed data. Differences in categorical variables were evaluated using the Chi-square test. For the comparison of BIVA and BIVA-Sp among subgroups, Mahalanobis distance was calculated, and Hotelling’s-T^2^ statistic was applied. Statistical significance was established at *p* < 0.05. All statistical analyses were performed using IBM-SPSS Statistics version 29.0 for Windows.

## 3. Results

A total of 95 subjects participated in the study (52 men [54.7%] and 43 women [45.3%]), with a mean age of 80.0 years (SD: 10.2; range: 61.0–105.5). The average length of stay in the institution was 15.9 years (SD: 20.7; range: 0–77.4). Among the participants, 54.7% (52) were diagnosed with dementia (major neurocognitive disorder), 21.1% (20) with schizophrenia, 16.8% (16) with intellectual disability, and 7.4% (7) with other psychiatric conditions.

The sample displayed a high degree of dependency. According to the Barthel Index classification, 20.0% (19) of the subjects were totally dependent, 46.3% (44) presented severe dependency, 27.4% (26) moderate dependency, and 6.3% (6) mild dependency; none of the participants were classified as independent. Regarding frailty, the FRAIL scale identified only 7.4% (7) of the patients as robust, while 34.7% (33) were categorized as pre-frail and 57.9% (55) as frail. Based on the MNA, all patients were classified as either malnourished [46.3% (44)] or at risk of MN [53.7% (51)], with a mean score of 16.8 points (4.0).

According to BMI, 27.4% (26) of participants were classified as at risk of MN, 16.8% (16) as malnourished, 5.3% (5) as overweight, and 3.2% (3) as obese, while 47.4% (45) had a normal weight. Visceral obesity, assessed through waist circumference, was present in 40% (38) of the sample. All BC variables (%FFM, %FM, FFMI, FMI, and ASMMI), as well as waist circumference, showed significant differences between men and women; no sex-related differences were observed in BMI.

Evaluation through the GLIM criteria revealed that 31.9% (30) of the subjects [36.5% (19) of men and 26.2% (11) of women] were classified as malnourished, with no significant sex-related differences. Obesity was present in 18.9% (18) of the study population [26.9% (14) of men and 9.3% (4) of women]. The prevalence of sarcopenia in the study sample was notably high [71.6% (68): 80.8% (42) in men and 60.5% (26) in women], whereas SO was identified in 12.6% (12) of the sample, all of whom were men (23.1% of the male participants). Significantly higher rates of obesity, sarcopenia, and SO were observed in men (*p* < 0.025).

Table 1 presents BC parameters estimated by conventional BIA by sex according to the presence or absence of MN, sarcopenia, obesity, and SO. Malnourished and sarcopenic men showed significantly lower values of FFMI and ASMMI compared to those without these conditions, whereas men with obesity and SO showed higher FMI than their non-obese counterparts. In men with SO, ASMM/W was significantly lower (23.7%) than in those without SO (29.6%; *p* < 0.001). In women, those with MN presented statistically significant differences in all BC variables. Similar trends to those observed in men were found among sarcopenic and obese women. No cases of SO were identified among female participants.

**Figure 1:**
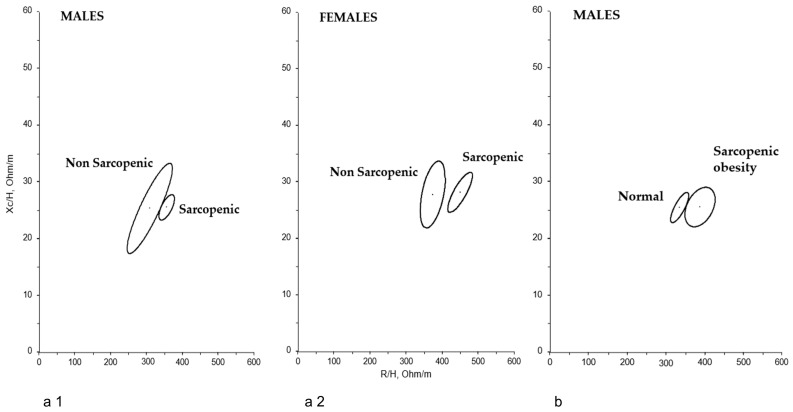
BIVA confidence ellipses for sarcopenic and non-sarcopenic men (*p* = 0.0068) (**a1**) and women (*p* < 0.0001) (**a2**); and (**b**) men with sarcopenic obesity versus the rest (*p* = 0.0005) (Hotelling’s-T^2^ test).

The classic BIVA identified significant differences in bioelectric behavior between men and women (*p* < 0.0001). However, BIVA did not reveal differences based on BMI categorization, visceral obesity, the presence of MN, or the presence of adiposity (in either men or women). Nonetheless, BIVA did allow for the differentiation of subjects based on the presence of sarcopenia (Figure 1(a1,a2)), both in men (*p* = 0.0068) and women (*p* < 0.0001), and based on the presence of SO in men (*p* = 0.0005) (Figure 1b)—no women had SO.

**Figure 2:**
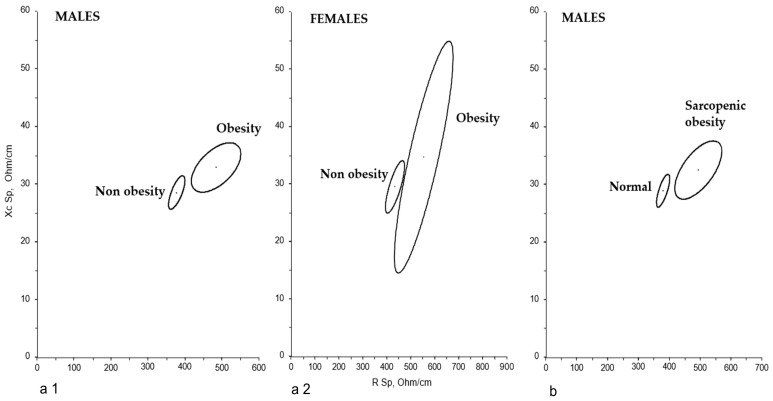
BIVA-Sp confidence ellipses for men (**a1**) and women (**a2**) based on the presence of adiposity (*p* < 0.0001 and *p* = 0.0218 in obese vs. non-obese men and women, respectively); and (**b**) men with sarcopenic obesity versus the rest (*p* < 0.0001) (Hotelling’s-T^2^ test).

Similarly, BIVA-Sp also identified differences in bioelectric behavior by gender (*p* = 0.0179); it did not reveal differences based on BMI categorization or the presence of MN in either men or women. However, unlike classic BIVA, BIVA-Sp did detect differences in both sexes regarding adiposity (*p* < 0.0001 men with obesity vs. non-obese; *p* = 0.0218 women with obesity vs. non-obese) (Figure 2(a1,a2)) and SO in men (*p* < 0.0001) (Figure 2b).

**Figure 3:**
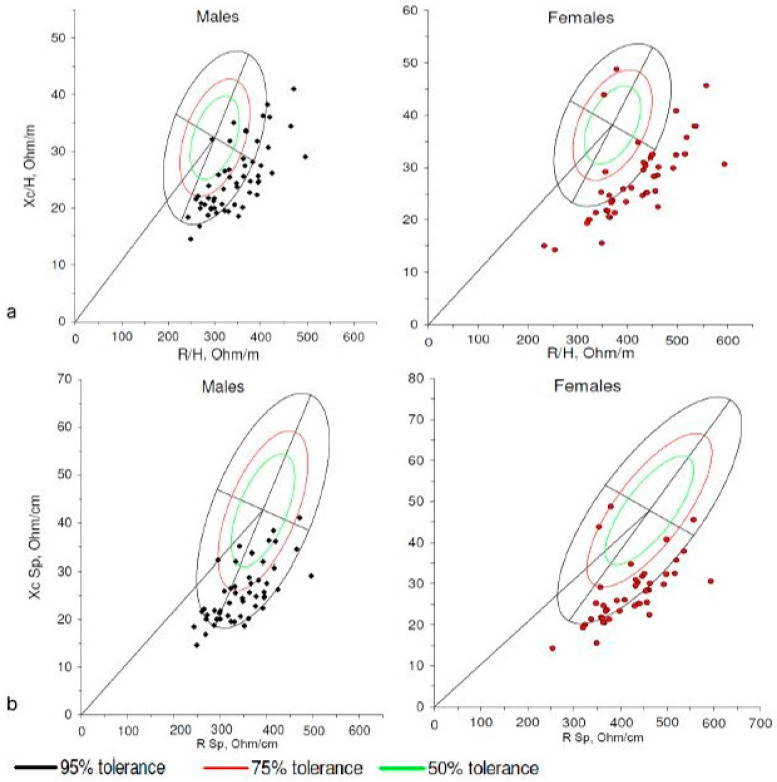
Tolerance ellipses of individual vectors for men and women in (**a**) BIVA and (**b**) BIVA-Sp. The black dots represent men and the red dots represent women.

Comparison of the sample with similar populations [39] revealed that our subjects are located in the lower right quadrant, with most falling outside the 75% tolerance ellipse, both when interpreting the bioelectrical data using BIVA and BIVA-Sp (Figure 3). Similar results were obtained when comparing them with other reference populations [9].

## 4. Discussion

The aim of this study was to evaluate the utility of BIA for detecting nutritional disorders associated with changes in BC in a group of institutionalized psychogeriatric patients. The results indicated that BC estimated by conventional BIA significantly differed according to nutritional disorders: lower FFMI and ASMMI in men and women with MN and sarcopenia, and higher FMI in patients with obesity and SO. Classic BIVA detected statistically significant differences based on sarcopenia and SO, while subjects with adiposity and SO showed different vector distributions in BIVA-Sp. Neither classic BIVA nor BIVA-Sp were able to differentiate subjects based on BMI categorization or MN.

Different modalities of BIA allow different interpretations of BC, body cell mass, and hydration status. Depending on the study’s goals and the population assessed, some modalities may be more suitable and accurate than others for detecting subtle changes in BC, hydration levels, or for short-term monitoring.

Classic BIA allows for the accurate estimation of different body compartments of FFM (FFM, total body water, SMM, ASMM), as long as predictive equations developed using similar BIA devices in populations with comparable physiological characteristics are used [8,41]. Classic BIA showed a significative reduction in FFM and ASMM in subjects with MN and sarcopenia, and an increase in FM in subjects with obesity, and SO (Table 1). Similar results have been obtained in various studies [42,43]. Malnourished women (11 subjects) also had significantly lower FM than non-malnourished women. Only 4 women were obese, and none had SO. It is interesting that men with SO did not show statistically significant differences in FFMI and ASMMI compared to non-sarcopenic obese men. This may be due to the fact that in the SOGLI criteria [4,35], the loss of ASMM is assessed in relation to weight (ASMM/W, which is significantly different in both groups), while in the EWGSOP2 criteria for sarcopenia [24], ASMM is normalized for height (ASMM/H^2^).

On the other hand, in a population as vulnerable as that of the present study, it is crucial to continuously assess nutritional status, as small changes in FM, FFM, or hydration status can occur over a short period of time and affect their clinical condition. However, several studies have documented that in older adults, classic BIA does not detect small changes in body compartments [14,15,16].

Nevertheless, classic BIVA enables detection of subtle BC changes in older adults [14,15,16]. Classic BIVA could be a useful method for monitoring hydration status: vector length on the RXc graph is inversely related to total body water, while PhA reflects tissue hydration. This method can detect shifts of less than 500 mL in tissue hydration with high accuracy (standard error: 2%), even in ill individuals [44], and may serve as a more reliable prognostic indicator than weight loss [8]. Moreover, BIVA also assesses nutritional status: variations in the vectors along the minor axis indicate changes in body cell mass in soft tissues, with higher amounts to the left [10,11]. BIVA differentiated subjects with sarcopenia from the rest (Figure 1(a1,a2)). The mean vector for sarcopenic subjects was positioned to the right of that for non-sarcopenic subjects (lower PhA) and was slightly longer, indicating lower body cell mass, higher extracellular to intracellular water ratio, and a slightly worse hydration status, which has already been documented in other studies [45,46,47,48]. However, some studies have only detected these differences in women, but not in older men [49], possibly due to the small sample size of the latter group. The distinctive bioelectric behavior observed in sarcopenic individuals has led some researchers to recommend the use of BIVA as an alternative to grip strength assessment in uncooperative subjects [50], such as psychogeriatric patients with cognitive impairment. BIVA detected similar behavior between men with SO and the rest (Figure 1b): slightly longer vector and lower PhA. Although classic BIVA has been barely used to study SO in older individuals, published studies on this topic have also found a lower PhA in these patients [51,52].

Interestingly, BIVA did not detect significant differences in the bioelectric behavior of malnourished men and women compared to non-malnourished individuals, unlike previous studies [48,53]. However, most used different diagnostic criteria for MN; only Guerrini’s group [48] applied GLIM, but studied a much younger population. In BIVA, malnourished individuals typically show reduced PhA and a short mean vector (lower right quadrant), as seen in most study participants. The nutritional status of our non-malnourished (per GLIM) but elderly and frail patients likely places them in the same quadrant, showing no significant differences from malnourished individuals. Other authors have also reported no BIVA-MN correlation in hospitalized patients [54].

Thus, as classic BIVA detects rapid hydration changes undetectable by conventional BIA, it is a valuable tool for monitoring nutritional and hydration status over time, without relying on predictive models [44]. It may also serve as a screening method: vectors outside the 75% tolerance ellipse indicate abnormal impedance, making predictive models inappropriate [15].

Classic BIVA also failed to distinguish subjects with altered FM, an issue resolved by BIVA-Sp. This modality accurately evaluates FM in overweight and obese individuals—who often present longer vectors—and assesses the extracellular-to-intracellular water ratio, even in older adults [12,17]. Thus, BIVA-Sp evaluates FM via vector length and muscle quantity/quality via PhA [55]. BIVA-Sp is also more effective than classic BIVA in identifying bioelectrical changes associated with psychofunctional and nutritional indicators in institutionalized older adults with dementia [18] and Alzheimer’s disease [56,57]. In the present study, both men and women with obesity, as well as men with SO, showed longer vectors and lower PhA than non-obese individuals, indicating higher FM and a lower ASMM/W. These findings are consistent with those reported by other authors in both younger and older populations [46,55]. Taken together, these results support the usefulness of BIVA-Sp for assessing FM, even in very vulnerable older adults. This modality may therefore represent a valuable tool for monitoring the nutritional status of individuals in whom changes in BC cannot be detected by other techniques, such as conventional BIA or anthropometry [57].

The study participants were old (mean age: 80 years), long-term-institutionalized, and characterized by high comorbidity, cognitive decline, dependency, and frailty, defining a highly vulnerable group at elevated risk of MN and sarcopenia, as reflected in the tolerance ellipses of Figure 3. As previously described [58], their vectors appeared in the lower-right quadrant compared with a reference older population, indicating lower PhA, reduced body cell mass, higher extracellular–intracellular water ratio (classic BIVA), and lower FM (BIVA-Sp). Some authors [24] propose using healthy young adults as the reference group; however, when ellipses from the Campa young adult population [59] were applied, 96.2% of men and 95.3% of women fell outside the 95% ellipse, with only minimal representation within the 75–95% or 50% ellipses. Similarly, using the BIVA-Sp young adult references [60], 96.2% of men and 95.3% of women were outside the 95% ellipse, again clustering mostly in the lower-right (men) and upper/lower-right areas (women). These results underscore the need for reference ellipses derived from healthy older adults, such as those employed in this study [9,39]. Nevertheless, the sample still clustered predominantly in the lower- and upper-right quadrants of older adult tolerance ellipses in both BIVA approaches, indicating low PhA—a pattern associated with MN, sarcopenia, frailty, normal aging [61,62], and cognitive impairment [63]. In this context, PhA acts as a general risk marker [61], rather than a disease-specific indicator. Nonetheless, BIVA modalities captured BC variations linked to prevalent nutritional disorders.

This study has several limitations, including a small sample size and its single-center design, as well as the presence of multimorbidity, which limits the generalizability of the findings to other institutionalized older populations. Although most participants were polymedicated (including diuretics) and exhibited age-related physiological decline (senescence), their clinical condition was stable. These characteristics are common among older adults with long-term stays in residential care facilities. Because participants were institutionalized, dietary intake and nighttime rest followed standardized protocols, ensuring consistent conditions across subjects, as did the procedures for conducting BIA (fasting state, rest, bladder emptying, temperature, time of day, posture, etc.). Moreover, patients with acute intercurrent conditions that could alter inflammatory status were excluded.

Further research is needed in larger and more diverse patient groups, including a wider range of psychogeriatric disorders and nutrition-related conditions, to enable meaningful subgroup analyses. A larger cohort would increase statistical power, potentially revealing bioelectrical differences between conditions such as obesity and SO, or across different psychogeriatric disorders, and would allow evaluation of responses to individualized nutritional interventions. It would also facilitate the development of reference ellipses for institutionalized older populations.

Additionally, no sample size calculation was performed in this study because its aim was not to test a specific hypothesis. However, the present work may serve as a basis for generating new hypotheses—for example, identifying cut-off points for adiposity or other clinical conditions (malnutrition, sarcopenia, SO) that could yield statistically significant confidence ellipses—and for determining the sample size required to detect such parameters with adequate statistical power in this population.

## 5. Conclusions

In an elderly, long-term-institutionalized population, with high comorbidity, cognitive decline, dependency, and frailty, classic BIVA detects changes in body cell mass and hydration (sarcopenia, SO), while specific BIVA identifies FM-related alterations like adiposity and SO.

The application of BIVA in these highly frail individuals enables the detection of changes in body composition and hydration status more easily, rapidly, and effectively than the conventional methods used in long-term care facilities, requiring minimal patient cooperation both at admission and during follow-up. When integrated into comprehensive geriatric assessment, this approach facilitates individualized nutritional management tailored to each patient’s clinical progression, potentially reducing morbidity, functional decline, and hospitalizations, thereby improving their quality of life.

## Figures and Tables

**Table 1:** Body composition in men and women according to different nutritional disorders and nutrition-related conditions with complex and multiple pathogenic backgrounds.

Conditions		FMI (kg/m^2^)		FFMI (kg/m^2^)		ASMMI (kg/m^2^)	
		Men	Women	Men	Women	Men	Women
Malnutrition	Present (19 M, 11 F)	5.8 (2.7)	6.7 (2.3) *	17.3 (1.6) *	13.0 (1.2) *	6.0 (0.7) *	4.8 (0.2) *
	Absent (33 M, 32 F)	6.0 (2.9)	8.5 (2.4)	16.1 (1.6)	14.3 (1.4)	6.5 (0.7)	5.3 (0.6)
Sarcopenia	Present (42 M, 26 F)	5.7 (2.7)	7.6 (2.1)	16.3 (1.3) *	13.1 (1.1) *	6.1 (0.5) *	4.8 (0.4) *
	Absent (10 M, 17 F)	6.8 (3.3)	8.8 (2.9)	19.0 (1.6)	15.3 (0.8)	7.2 (0.7)	5.8 (0.4)
Obesity	Present (14 M, 4 F)	9.6 (1.8) *	11.5 (0.7) *	17.1 (2.0)	14.6 (0.9)	6.4 (0.8)	5.4 (0.3)
	Absent (38 M, 39 F)	4.5 (1.6)	7.7 (2.3)	16.8 (1.6)	13.9 (1.5)	6.3 (0.7)	5.2 (0.6)
Sarcopenic obesity	Present (12 M, 0 F)	9.6 (1.9) *	—	16.6 (1.7)	—	6.2 (0.7)	—
	Absent (40 M, 43 F)	4.8 (1.9)	8.1 (2.5)	16.9 (1.7)	14.0 (1.5)	6.4 (0.7)	5.2 (0.6)

* *p* < 0.05 present vs. absent (Student’s *t*-test for independent samples or Mann–Whitney U-test). M: males; F: females; FMI: fat mass index; FFMI: fat-free mass index; ASMMI: appendicular skeletal muscle mass index.

## Data Availability

The data presented in this study are available on request from the corresponding author due to privacy restrictions.

## Abbreviations

The following abbreviations are used in this manuscript:

ASMM	Appendicular skeletal muscle mass
ASMMI	Appendicular skeletal muscle mass index
ASMM/W	Appendicular skeletal muscle mass relative to body weight
BC	Body composition
BIA	Bioelectrical impedance analysis
BIVA-Sp	Specific bioelectrical impedance vector analysis
BMI	Body mass index
DXA	Dual-energy X-ray absorptiometry
EWGSOP2	European Working Group on Sarcopenia in Older People 2
FFM	Fat-free mass
FFMI	Fat-free mass index
FM	Fat mass
FM%	Fat mass percentage
FMI	Fat mass index
GLIM	Global Leadership Initiative on Malnutrition
ICD-11	International Classification of Diseases, 11th Revision
MNA	Mini Nutritional Assessment
PhA	Phase angle
R	Resistance
R/H	Resistance standardized by height
Rsp	Specific resistance
SD	Standard deviation
SEGG	Spanish Society of Geriatrics and Gerontology
SENPE	Spanish Society of Parenteral and Enteral Nutrition
SMM	Skeletal muscle mass
SO	Sarcopenic obesity
SOGLI	Sarcopenic Obesity Global Leadership Initiative
Xc	Reactance
Xc/H	Reactance standardized by height
Xcsp	Specific reactance
